# Systems biology approaches to unravelling neonatal sepsis and potential for precision diagnostics and therapeutics

**DOI:** 10.3389/fphar.2026.1778409

**Published:** 2026-04-30

**Authors:** Pearl Park, Negin Ketabchi, Breanna B. Raymond, Andy Y. An, Erica Acton, Constantin R. Popescu, Pascal M. Lavoie, Amy H. Y. Lee

**Affiliations:** 1 Department of Molecular Biology and Biochemistry, Simon Fraser University, Burnaby, BC, Canada; 2 British Columbia Children's Hospital Research Institute, Vancouver, BC, Canada; 3 Prevention of Organ Failure (PROOF) Centre of Excellence, University of British Columbia, Vancouver, BC, Canada; 4 Department of Microbiology and Immunology, The University of British Columbia, Vancouver, BC, Canada; 5 Department of Pediatrics, Université Laval, Québec, QC, Canada; 6 Department of Pediatrics, University of British Columbia, Vancouver, BC, Canada

**Keywords:** biomarkers, immunology, neonates, pathophysiology, precision medicine, sepsis

## Abstract

Sepsis is a major cause of global neonatal mortality, affecting 1.3 to 1.9 million neonates worldwide. Accurate diagnoses and effective treatment of sepsis during the onset is essential to improve outcomes. Current efforts to develop sensitive molecular biomarkers, however, are limited by the lack of accurate diagnostic tests, particularly the lack of sensitive viral testing or blood culture methods with low blood inoculums from small neonates. Consequently, antibiotic treatments are often misused, contributing to antimicrobial resistance among neonates exposed to unnecessary antibiotics. To improve the management of neonatal sepsis, a thorough understanding of early neonatal immune development and the pathophysiology of the disease is required. In this review, we summarize the current understanding of the newborn’s immune system based on different gestational ages and describe its influence on molecular mechanisms characterizing neonatal sepsis. We also focus on current endeavors for neonatal sepsis diagnosis and new approaches using omics-based biomarkers, highlighting their limitations and future directions for clinical applications. Lastly, we will discuss how utilizing systems immunology provides mechanistic insights and potential avenues towards therapeutics.

## Introduction

Neonatal sepsis is characterized by a dysregulated and life-threatening immune response to severe infection in neonates (infants less than 28 days old) (Simonsen et al., 2014). Globally, the greatest burden of disease occurs in low- and middle-income countries (LMICs) (Milton et al., 2022). Sepsis most commonly originates from bacterial or viral infections, and occasionally from fungi and parasites (Cortese et al., 2016). This review will focus on bacterial sepsis as these are the predominant cases (Saha et al., 2018; Stoll et al., 2020). Depending on the time of symptom onset, sepsis can be classified into early-onset sepsis (EOS) or late-onset sepsis (LOS). EOS occurs generally in the first 72 h of life (up to 7 days after birth for full term infants) (Bulkowstein et al., 2016), most commonly from a perinatal infection that is vertically transmitted from the mother (e.g., chorioamnionitis or maternal Group B streptococcal colonization) before or at the time of birth (Simonsen et al., 2014). In contrast, LOS is acquired from the community or hospital settings, and symptoms typically appear after 7 days, though can appear earlier in premature and very low birth weight (VLBW) neonates after 72 h, when compared to term neonates (Simonsen et al., 2014).

Stark differences in sepsis etiology and health outcomes can be observed among neonates with different gestational ages (GA) (Afonso et al., 2023). Preterm neonates (born earlier than 37 weeks GA) have increased susceptibility to infection due to specific characteristics of their immune system. Even though neonatal sepsis can impact both term and preterm neonates, a disproportionately higher EOS incidence is observed in preterm infants (Stoll et al., 2020). When compared to term neonates, the incidence of EOS is 30-fold higher in extremely preterm neonates (GA 22–28 weeks) and 11-fold higher in very preterm neonates (GA 29–33 weeks) (Stoll et al., 2020). Since these neonates are hospitalized at birth, the use of invasive devices such as intravascular catheters and mechanical ventilation may increase the risk of sepsis from nosocomial infections, with pathogens such as coagulase-negative *Staphylococcus*, *Staphylococcus aureus, Enterococcus*, *Escherichia coli*, *Pseudomonas aeruginosa*, *Klebsiella pneumoniae,* and fungi (Sohn et al., 2001). Gestational age is therefore a crucial factor determining sepsis susceptibility, in part, due to its influence on the maturity of the immune system.

Neonatal sepsis outcomes depend on early recognition of the disease, typically hampered by the non-specific nature of clinical signs (Wynn et al., 2014; Molloy et al., 2020), including tachycardia/bradycardia, temperature instability, respiratory distress, lethargy, poor feeding, vomiting, and jaundice (Singh et al., 2023). These non-specific signs occur in both bacterial and viral infections, often leading to deaths recorded as ‘possible serious infections’ with causes unknown (Saha et al., 2018). The non-specificity of sepsis signs increases the risk of misdiagnosis; therefore there is a low threshold for clinicians to initiate a full septic workup (including blood cultures and often a lumbar puncture for cerebrospinal fluid [CSF] culture) and initiate empiric antibiotic treatment for suspected bacterial sepsis or empiric antiviral treatment when viral sepsis is suspected (Simonsen et al., 2014). In cases of suspected viral sepsis, molecular diagnostic tests using PCR on different samples are needed depending on the viral pathogens. For example, CSF or blood is needed for potential herpes simplex virus infection, while nasopharyngeal swabs are used for respiratory pathogens such as respiratory syncytial virus, influenza virus and human metapneumoviruses, or stool and urine for culturing enterovirus (Burstein et al., 2024).

The gold standard for bacterial sepsis diagnosis is identifying a positive pathogen growth from a sterile-site culture (typically blood) in the presence of suggestive clinical signs (Molloy et al., 2020). It takes anywhere from several hours and up to 2–3 days to culture different pathogens, resulting in further delay in definitive diagnosis (Iroh Tam and Bendel, 2017). While these cultures are pending, neonates are generally empirically covered by broad-spectrum antibiotics, as a delay in treatment results in a higher mortality rate (Lavoie et al., 2019; Giannoni et al., 2022). However, over 70% of sepsis-causing Gram-negative bacteria in LMICs are already resistant to front-line ampicillin and gentamicin, limiting empiric antibiotic choices (Thomson et al., 2021). Prolonged antibiotic use also presents its own risks, and the adverse consequences of unnecessary antibiotics use are well documented (Lavoie et al., 2019; Ramasethu and Kawakita, 2017). For example, a systematic review and meta-analysis showed prolonged antibiotic resistance for up to 12 months in individuals who received a first treatment in primary care (Costelloe et al., 2010). In pediatrics, another study of VLBW preterm neonates showed that administration of empiric antibiotic therapy was associated with a 1.24 times greater risk for LOS, necrotizing enterocolitis (NEC), and death (Cantey et al., 2018). This warrants judicious use of empiric antibiotics, namely, balancing between having sufficient coverage to treat a yet-unidentified pathogen while also preventing long-term health outcomes from microbiome dysbiosis and the growing threat of antimicrobial resistance (Cantey et al., 2018). A better understanding of who is at risk could help adopt a more judicious use of antibiotics. For this, some basic knowledge of host immunology is required, taking into consideration various factors that influence susceptibility to neonatal sepsis, with gestational age being the most significant. Preterm neonates are particularly prone to sepsis compared to term neonates. Here, we review the early postnatal developmental stages of term versus preterm infants, which will establish a general understanding of the different immune responses between these gestational age groups towards general infection, and more specifically, in response to sepsis. Detailed reviews of fetal immune development during gestation are discussed elsewhere (Kan et al., 2016; Kollmann et al., 2017; Donald and Finlay, 2023). Though viral pathogens are known to cause neonatal sepsis, this review will predominately focus on how systems immunology improves our understanding of neonatal immune responses to bacterial sepsis, informs the development of molecular biomarkers, provides potential therapeutic opportunities and future directions for this field.

## Immune responses of healthy term and preterm neonates

Immune maturation during early life is complex and requires balancing between tolerating commensal microbial colonization while defending against infectious pathogens. This balance needs to be achieved also during a critical period of high metabolic demands in the growing organism (Kollmann et al., 2017; Conti et al., 2020). Differences in gestational age further contribute to this complexity, with younger gestational ages making faster growing neonates more vulnerable to disease.

### Dynamic development of term and preterm neonatal immunity

Unlike the typical steady state seen in adult immune systems, the fetal and neonatal immune system undergoes dynamic, yet structured development phases in the first few years, with potential long-term health implications when this maturation is perturbed (Olin et al., 2018; Lee et al., 2019; Ezinmegnon et al., 2022). In a Swedish cohort of 100 infants (50 very preterm with GA < 30 weeks and 50 term infants ≥37 weeks), longitudinal sampling of very preterm and term neonatal whole blood showed that while the immune cell populations and plasma proteins differed at birth, they eventually converged onto a shared trajectory (Olin et al., 2018). Preterm births are associated with inflammatory processes or infections (Humberg et al., 2020), reflected in a pro-inflammatory signature from plasma proteomics of preterm cord blood. This was characterized by the expression of interleukin(IL)-8/C-X-C motif chemokine (CXCL)-8 (a neutrophil chemoattractant secreted by newborn T cells) and CXCL-11 (a T cell chemoattractant secreted mainly by monocytes) in preterm cord blood compared to term cord blood (Wang et al., 2024). Cord blood from term infants typically showed higher neutrophil counts compared to preterm cord blood, with neutrophil levels positively correlated to gestational age (Olin et al., 2018).

To provide a deeper understanding of how term and preterm infants may develop over time, peripheral whole blood samples were taken from this cohort at 1, 4, and 12 week(s) of life (Olin et al., 2018), as well as cord blood at birth. Using a topological data analysis of plasma proteomics and immune cell populations to construct a landscape model, Olin et al. demonstrated that while preterm and term cord blood samples were initially distinct based on proteomic and transcriptomic changes, there was a rapid convergence between the postnatal immune development of term versus preterm infants, with samples at 3 months of age intermixing. The convergence of the preterm versus term immunity was largely driven by a reduction in neutrophils, an increase in CD4^+^ and CD8^+^ T cell proportions over postnatal age, and convergence of IL8/CXCL8 levels. The dynamic changes observed in both preterm and term immune cell phenotypes and composition were not seen in the relatively stable phenotypes in adults over time (Olin et al., 2018). Thus, it appeared that despite several differences in gestational age, there was an almost “pre-programmed” path of immune development that neonates followed, likely with microbial exposure as its main driver, highlighting the remarkable ability of neonates to adapt and respond to their environment (Olin et al., 2018). Despite this convergence, preterm infants remain at high risk of neonatal sepsis. An explanation for this may be the initial differences observed before the convergence of immune phenotypes and composition, but as mentioned by Olin et al., immunological factors were not the sole contributors to immune responses and further research would be required to understand other contributing factors to immune responses that render preterm neonates more susceptible (Olin et al., 2018).

This dynamic immune development, termed “ontogeny”, was further refined in a multi-omics characterization of two distinct cohorts of full-term neonates from the Gambia and Papua New Guinea (Lee et al., 2019). Using less than 1 mL of newborn peripheral venous blood, longitudinal multi-omics analyses including immune phenotyping, whole blood transcriptomics, plasma proteomics and plasma metabolomics, were assessed over the first week of life (Lee et al., 2019). In these term infants, CXCL-10, IL-17A, macrophage-derived chemokine (MDC), and interferon (IFN)γ increased, while IL-10, Chemokine C−C motif ligand (CCL)-5, granulocyte colony stimulating factor 2 (G-CSF2), and IL-6 decreased over the first week of postnatal life. The trends observed with IL-17A and IL-10 were consistent with those reported in Olin et al., despite the methodological and cohort differences. In term infants, there was a progressive decrease in proportions of neutrophils, basophils, plasmacytoid dendritic cells, and natural killer cells during the first week of life while myeloid dendritic cell proportions increased over time (Lee et al., 2019). Whole blood transcriptomics, plasma proteomics and plasma metabolomics further highlighted the dynamic ontogeny observed during the first week of life. Using three different multi-omics integration analysis strategies, interferon signaling, complement cascade and granulocyte function were identified as specific pathways that drove molecular ontogeny over the first week of life. By harnessing the power of longitudinal studies and maintaining consistent timing of samples during comparisons, both studies greatly contributed to understanding neonatal immune responses, particularly in the first few weeks of life. The dynamic immune baseline and initial differences in term and preterm neonatal immunity during the early postnatal period may explain the observed differences in innate, adaptive and passive immune responses toward infection. We explore these differences in further detail below (Table 1).

**TABLE 1:** Comparing the innate, adaptive, and passive immune responses between preterm and term neonates.

Immunity types	Preterm immunity compared to full-term immunity
Innate	• Lower levels of APPs on skin (Battersby et al., 2016; Strunk et al., 2009) • Generally, lower numbers of classical monocytes, greater intermediate monocytes (Anderson et al., 2021; De Biasi et al., 2023) • Slower NET formation rate and smaller sizes (Lipp et al., 2017)
Adaptive	• Greater skew towards Th2 response (Qazi et al., 2020; Semmes et al., 2020) • More transitional B cells (Anderson et al., 2021)
Passive	• Lower levels of maternal IgG (Pou et al., 2019) • Similar IgG dependent neutralization and phagocytosis (Pou et al., 2019)

## Immune responses of term and preterm neonates to sepsis

**FIGURE 1:**
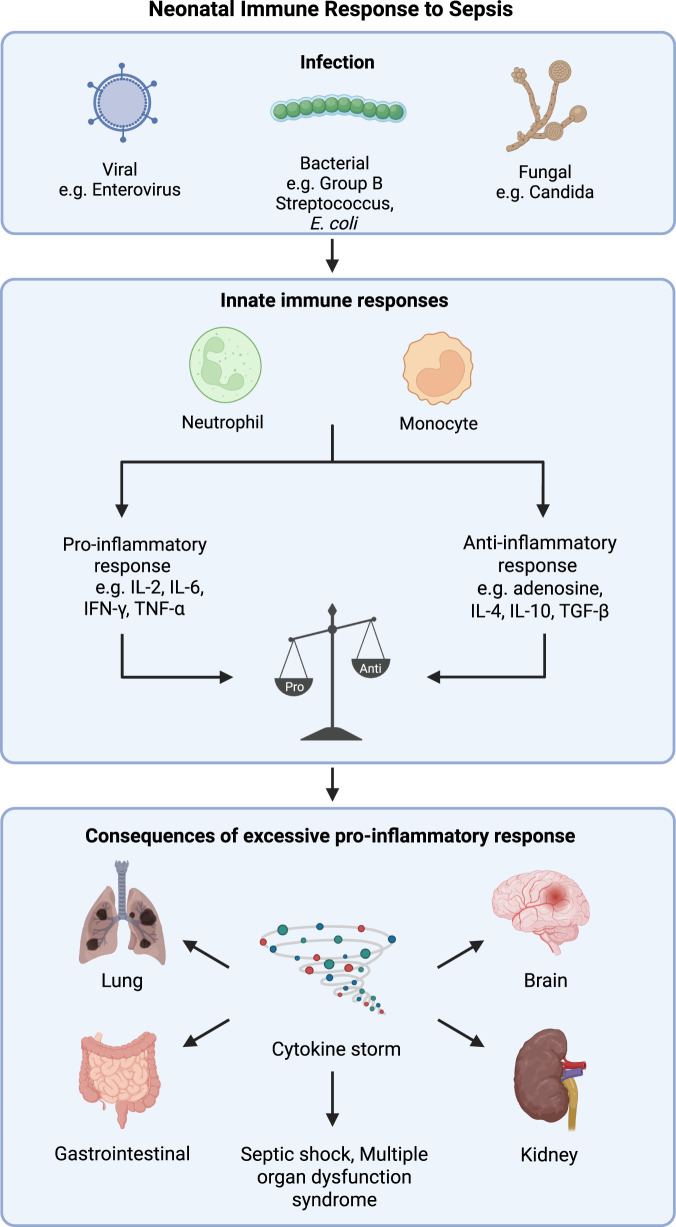
Neonatal immune response to sepsis. Sepsis originates from a viral, bacterial or fungal infection that triggers immune cells to initiate pro- and anti-inflammatory responses. Excessive cytokines from the proinflammatory response can result in a cytokine storm as well as septic shock and multi-organ dysfunction syndrome. Image created in BioRender. Lee, A. (2026) https://BioRender.com/p85p264.

During an infection, the recognition of pathogens by innate immune cells triggers an immune response aimed at clearing the pathogen (Wynn and Wong, 2017). However, in neonatal sepsis, this initial immune response becomes dysregulated with both excessive inflammatory and anti-inflammatory responses (Wynn and Wong, 2017; Ng et al., 2003). This dysfunctional release of cytokines, known as a cytokine storm, can alter the advantageous cytokine response into exaggerated and harmful inflammation. This can progress to septic shock, characterized by a decreased perfusion to organs, ultimately leading to multiple organ dysfunction syndrome (MODS), which is often fatal (Figure 1) (Wynn and Wong, 2017).

### Immune deficiencies that predispose preterm neonates to sepsis

Preterm neonates, as outlined earlier, have deficiencies in their innate immune responses compared to term neonates possibly due to immune maturation occurring later in gestation (Kan et al., 2016; Tissieres et al., 2012; Sharma et al., 2014), which likely predisposes them to developing neonatal sepsis. Neutrophils play a crucial role in the inflammatory response during sepsis, being one of the first and primary responders to infections (Smith et al., 2014). Pattern Recognition Receptor (PRR)-dependent innate responses become active late in the third trimester of gestation, possibly due to mTOR-mediated metabolic inhibition (Kan et al., 2018; Michalski et al., 2024). Neonatal neutrophils also show several functional deficiencies, including reduced levels of bactericidal/permeability-increasing proteins against Gram-negative bacteria, which may increase the risk of Gram-negative sepsis, such as *E. coli* (Levy et al., 1999). Younger gestational age negatively influences neutrophil formation and function (Lawrence et al., 2017), as well as their migration to effector sites in response to microbial products (Raymond et al., 2017); this may account for the elevated risk of neonatal sepsis (Tissieres et al., 2012). Whole blood transcriptomics revealed significant differences in gene expression between preterm and term neonates, including deficiencies in IL-1, inducible Nitric Oxide Synthase (iNOS) signaling, and the inflammasome pathway (Raymond et al., 2017). In a septic murine model, neonatal neutrophils showed increased neutrophil extracellular traps (NETs) compared to adult neutrophils. This excessive NET production, termed NETosis, could play a role exacerbating the inflammatory response to sepsis in neonates, leading to more multi-organ dysfunction (Colon et al., 2019). This was supported by work done by Nguyen et al. which demonstrated that preterm pigs and mice infected with *S. epidermidis*, as well as septic preterm human neonates, had significantly higher levels of circulating cell-free DNA (cfDNA) —a marker of NETs released by activated neutrophils during the process of NETosis—compared to healthy controls during LOS (Nguyen et al., 2017).

Similar to neutrophils, monocyte function is also reduced in younger GA. In preterm neonates, the functional immaturity of monocyte activation pathways, necessary for mounting a sufficient cytokine response to bacteria, may contribute to their susceptibility to sepsis with commensal bacteria (Strunk et al., 2012). Moreover, the phagocytic abilities of monocytes collected from cord blood in VLBW infants appeared to be diminished during EOS (Hallwirth et al., 2002). These deficiencies in immune functions in preterm neonates likely explain the increased risk of sepsis in this population.

### Concurrent pro- and anti-inflammatory responses in septic preterm and term neonates

The immune responses of preterm and term neonates to sepsis are marked by immune differences that may impact their susceptibility and clinical outcomes. As mentioned previously, functional differences in neutrophil and monocyte function related to GA may predispose preterm neonates to sepsis (Collins et al., 2018). Both preterm and term neonates experience a dysregulated balance of pro- and anti-inflammatory cytokines during sepsis that can result in inflammatory responses, organ dysfunction and increased mortality. Therefore, maintaining a harmonious balance between the pro- and anti-inflammatory responses is crucial to prevent excessive inflammatory damage.

In a cohort of septic preterm neonates, both pro-inflammatory (IL-2, IL-6, IFN-γ, Tumor Necrosis Factor (TNF)-*α*) and anti-inflammatory (IL-4 and IL-10) cytokines were detected during initial sepsis evaluation, and at 24 and 48 h later (Ng et al., 2003). Most prominently, IL-6 and IL-10 were elevated by 35- and 22-folds, respectively, compared to non-infected preterm infants (Ng et al., 2003). This dual cytokine response was also observed in transcriptomic work done by Ng et al., with a total of 1317 differentially expressed genes in whole blood between septic and healthy very preterm infants. These genes were associated with pathogen recognition, cytokine signaling, and immune and hematological regulation (Ng et al., 2020). Both pro-inflammatory and anti-inflammatory responses were detectable at the onset of LOS, with elevated expression of IFN-α/β, IFN-γ, IL-1 and IL-6, as well as IL-10, SOCS1 and SOCS3 (negative regulators of IFN signaling pathways), among septic very preterm infants compared to non-infected neonates (Ng et al., 2020). In term neonates, several studies showed significantly increased serum levels of IL-1β, IL-6, IL-8, and TNF-α in septic neonates compared to healthy newborns (Chen et al., 2022; Kurt et al., 2007; Leal et al., 2019).

Interestingly, Segura-Cervantes et al. reported that septic preterm neonates (32–36 weeks GA) exhibit elevated levels of pro-inflammatory cytokines (TNF-α, IFN-γ, and IL-6) compared to very preterm septic neonates (<32 weeks GA) (Segura-Cervantes et al., 2016). However, anti-inflammatory cytokines (IL-4 and IL-10) were elevated during sepsis in both preterm and very preterm neonate groups compared to samples taken a week before and after sepsis diagnosis (Segura-Cervantes et al., 2016). These findings suggest again that the neonatal immune response is impacted by GA, with preterm infants showing a more robust proinflammatory cytokine response that is less pronounced in very preterm neonates. Ye et al. found that IL-10 levels were significantly increased in a cohort of 420 preterm and term septic neonates compared to a control group consisting of healthy neonates as well as neonates with jaundice or enterovirus infection, suggesting that GA may be inversely associated with more robust pro- and anti-inflammatory responses (Ye et al., 2017; Hibbert et al., 2018).

Almost in sync with the pro-inflammatory response, anti-inflammatory molecules such as adenosine (Levy et al., 2006), IL-4 and IL-10 from monocytes, and transforming-growth-factor-β (TGF-β) from T cells, skew the immune system towards T helper (Th) 2 response which counters the Th1 proinflammatory response and helps restore homeostasis (Wynn, 2016) (Table 1). Depending on the cytokines produced, naïve CD4^+^ T cells differentiate into Th1 or Th2 helper T cells (Wynn, 2016). For instance, Th1 cells are produced from exposure to IFN-γ, and IL-12, and TNF-α, while Th2 cells arise in the presence of IL-4, IL-5 and IL-13 cytokines (Wynn, 2016). However, prolonged anti-inflammatory responses may lead to further immunosuppression, making subjects more susceptible to secondary infections, such as fungal infections (Hibbert et al., 2018; El-Sahrigy et al., 2022). As such, any exacerbated response from either end of the immune spectrum can be detrimental to septic neonates, with additional damaging effects on vital organs such as the lungs and brain (Hibbert et al., 2018), which are worsened in preterm compared to term neonates (Marchant et al., 2015; Chang et al., 2011).

One potential explanation for a dual pro- and anti-inflammatory response during neonatal sepsis in both term and preterm neonates (Wynn and Wong, 2017; Kurt et al., 2007; Hibbert et al., 2018; Prashant et al., 2013) is the release of anti-inflammatory cytokines as a response to profound inflammation. Proinflammatory cytokines are crucial in activating immune cells to clear infections and resolve sepsis. However, severe inflammation can lead to endothelial dysfunction causing capillary leakage and further progress to septic shock, multiple organ failure, and death (Wynn and Wong, 2010). Endothelial dysfunction during sepsis is influenced by dysfunction of the angiopoietin and tyrosine-protein kinase receptor (Ang/Tie) signaling axis, which is diminished in both preterm and term septic neonates (Fidanza et al., 2024).

## Neonatal sepsis diagnostics

A molecular diagnostic marker for sepsis would be an invaluable tool to overcome the challenges of blood cultures and the lack of specific clinical symptoms. Although there has been extensive research on adult sepsis diagnostics, the adult immune system is functionally distinct from the neonatal immune system, with early life developmental changes being more dynamic in nature (Olin et al., 2018; Lee et al., 2019). We review the limitations to current microbial and clinical approaches and explore the future direction of utilizing molecular markers for earlier detection of neonatal sepsis.

Neonatal sepsis is diagnosed microbiologically or clinically. Blood culture is the gold standard for diagnosis and to inform treatment, as confirming the systemic nature of the infection, the causative pathogen, and its susceptibilities are essential for directed antibiotic therapy (Shane et al., 2017). However, blood cultures have relatively long turnaround times and lack sensitivity in small neonates in whom limited blood volumes are available for testing (Zea-Vera and Ochoa, 2015; Celik et al., 2021). Cultures are also prone to false positive results due to contamination from skin commensals, such as coagulase-negative *Staphylococcus* (Iroh Tam and Bendel, 2017). The risk of contamination is associated with a lack of adherence to aseptic techniques during sample collection (Buttery, 2002). Increasing the volumes of sampled blood increases sensitivity, but this is often not feasible in a critically ill newborn, particularly those who are preterm (Keij et al., 2020). The perceived lack of sensitivity of these tests results in prolonged empiric antibiotics in neonates with clinical signs of sepsis despite negative blood cultures (Celik et al., 2021). These culture-negative sepsis-suspected neonates are considered to have “clinical sepsis” (Polin et al., 2012).

The neonatal systemic inflammatory response syndrome (SIRS) criteria (Iroh Tam and Bendel, 2017) are a key factor in the clinical diagnosis of sepsis in older age groups. It requires the inclusion of at least two of four parameters (abnormal leukocyte counts, temperature, heart rate, or respiratory rate), with at least one of which must be abnormal temperature or leukocyte count (Wynn, 2016). However, the standard SIRS criteria lack sensitivity and have poor specificity in neonates as there are several non-septic conditions that can also result in SIRS-like features (Chang et al., 2011; Hofer et al., 2012; Poggi et al., 2023; Lavoie et al., 2010). The recent development of the neonatal Sequential Organ Failure Assessment (nSOFA) may better diagnose and predict mortality in VLBW neonates, particularly in high income countries, compared to the standard SIRS criteria (Poggi et al., 2023). Nevertheless, these novel SOFA criteria are currently not widely used in clinical practice and are not applicable in LMICs as the score incorporates diagnostic test results and interventions that are often unavailable in those settings (Wynn and Polin, 2019; Fleiss et al., 2021; Akinseye et al., 2023). Another option is the Kaiser Permanente EOS calculator, which is only used for neonates (>34 weeks GA) at risk of EOS, to guide the need for antibiotics. This calculator has been increasingly used to reduce unnecessary antibiotic administration (Kuzniewicz et al., 2017). Nevertheless, a recent meta-analysis revealed that the calculator demonstrates frequent false negatives in settings outside Europe and North America, suggesting that further adaptations are necessary for LMIC settings (Pettinger et al., 2020). Lastly, laboratory tests such as cell counts lacks both sensitivity and specificity for neonatal sepsis diagnosis. In a cohort of 37,826 preterm and term neonates with LOS admitted to the neonatal intensive care unit, complete blood counts (CBC) were measured, and high absolute neutrophil counts (>17,670/mm3), as well as high immature-to-total neutrophil ratios (>0.2), were shown to be associated with LOS (Hornik et al., 2012). However, because cell proportions show important changes by post-natal age and developmental period (Olin et al., 2018), it has been difficult to define specific threshold broadly applicable in clinical practice.

Overall, the limitations of blood cultures and the use of clinical signs of sepsis, mandates alternative approaches to sepsis diagnosis in neonates. This has sparked significant interest in diagnosing sepsis by measuring biological changes in samples such as blood, urine, and stool, using systems biology approaches (Ng et al., 2018; Iregbu et al., 2022). Indeed, approaching sepsis diagnosis from this perspective has the potential to overcome sensitivity issues, since measuring biological responses to sepsis can indicate the presence of the disease even when the pathogen is unculturable. The future of neonatal sepsis diagnostics likely will involve assessing not the pathogen, but the host response to the pathogen as well as considering developmental age, to find sensitive and specific biomarkers that are not constrained by the limitations of our current diagnostic tools.

### Molecular diagnostic markers

There is extensive interest in diagnostic molecular biomarkers for neonatal sepsis that reflect the host response, which have mainly been focused on cytokines and acute-phase reactants, such as C-reactive protein (CRP) and procalcitonin (PCT) (Chiesa et al., 2003). Several cytokines are elevated in sepsis, as discussed earlier, including pro-inflammatory cytokines such as IL-1, IL-1β, IL-2, IL-6, IL-8, IFN-γ, TNF-α, and IFN-α/β as well as anti-inflammatory cytokines including IL-4, IL-10, SOCS1, and SOCS3. CRP levels increase in blood in response to inflammation, peaking around 24–48 h, making it a relatively late marker of neonatal infection (Celik et al., 2021). This delay is a disadvantage of CRP, which makes this biomarker unreliable for early detection of neonatal sepsis (Celik et al., 2021; Brown et al., 2020). On the other hand, PCT has a more rapid response than CRP with a peak in elevation within 6–8 h of exposure to bacteria (Celik et al., 2021). This makes PCT an interesting marker for earlier diagnosis of bacterial neonatal sepsis (Celik et al., 2021). However, regular monitoring of blood samples for preterm neonates at the neonatal intensive care units is still required to effectively track disease progression (Ezinmegnon et al., 2022; Persad et al., 2021). Currently, no single available host immune biomarker has shown clear superiority over clinical assessment combined with conventional microbiologic testing for diagnostic purposes (Iroh Tam and Bendel, 2017). One promising avenue is to combine several biomarkers in one test. However, to find appropriate combinations, there needs to be an unbiased approach of interpreting large amounts of data. This is why “omics” studies have recently emerged to tackle the limitations of traditional clinical sepsis biomarkers (Iregbu et al., 2022).

### Omics markers

Omics refers to systems biology approaches that utilize large-scale data along with bioinformatic analyses to help advance complex biological processes. These high-throughput technologies include but are not limited to transcriptomics, proteomics, and metabolomics, with each field using different tools to study specific molecules of the septic response. Through an unbiased approach to interpreting high-throughput datasets (e.g., assaying all the gene products in the transcriptome), which has been greatly facilitated by advances in artificial intelligence and machine learning models, omics studies can substantially expand the repertoire of possible biomarkers. This allows for characterizing groups of omics markers instead of single molecules (e.g., PCT or CRP) to improve diagnostic accuracy. Omics approaches allow scientists to uncover novel combinations of biomarkers without the limitations of traditional biomarker approaches, while leveraging the growing arsenal of advanced machine learning tools and multi-omics integration strategies. For a more detailed review on omics biomarker development for neonatal sepsis, the authors suggest (Langley and Wong, 2017; Ng et al., 2018).

Advances in omics sample processing protocols now allow for many different omics types to be assayed in small blood volumes well below 1 mL (Lee et al., 2019), which is critical when dealing with neonates, either peripheral or cord blood. In addition to blood, other sample sources include urine and even amniotic fluid. Distinguishing sepsis from healthy controls or non-sepsis illness with similar symptoms is made possible through omics signatures comparing differentially abundant gene expression, proteins or metabolites in these distinct cohorts. Once biomarkers have been found through these high-throughput omics analyses, the premise is that these biomarkers would be implementable at the bedside. Proteins and metabolites can be measured with enzyme-linked immunosorbent assays (ELISA) while gene transcripts can be detected through real time quantitative polymerase chain reactions (qRT-PCR). The results from these bedside tests would be imputed into a trained algorithm to determine the diagnosis.

For proteomic biomarkers, potential biomarkers include cytokines (Ezinmegnon et al., 2022; Chen et al., 2022) and other immune-related proteins (Chatziioannou et al., 2018; Sylvester et al., 2014), as well as proteins seemingly unrelated to the immune response (Sylvester et al., 2014). Metabolomics biomarkers have focused primarily on sugars, amino acids (Dessì et al., 2014; Fanos et al., 2014) and lipids (Wang et al., 2023). Finally, transcriptomics studies have yielded gene signatures with potential diagnostic use using many genes related to the immune response (Smith et al., 2014; Ng et al., 2020; An et al., 2024; Al Gharaibeh et al., 2025). These omic analyses can help differentiate sepsis from other diseases with overlapping symptoms (e.g., NEC) (Sylvester et al., 2014; Ng et al., 2010), and among bacterial pathogens, between Gram-positive vs. Gram-negative bacterial sepsis (Ng et al., 2018; Langley and Wong, 2017; Cernada et al., 2022). Omics analyses could also help distinguish responses produced between viral and bacterial sepsis. For example, whole-blood transcriptomic analyses in a pediatric cohort aged 1 month to 17 years with suspected sepsis, used a forward selection partial least squares classifier to develop a ten-gene disease class signature that can differentiate bacterial, viral versus non-infectious causes (Schlapbach et al., 2024). In adult sepsis, TriVerity (Liesenfeld et al., 2025) is an FDA-approved rapid blood test that can determine the likelihood of bacterial or viral infection on patients suspected with sepsis presenting to the emergency departments. Using a set of 29 host immune genes initially identified from transcriptomics studies (Sweeney et al., 2015; Sweeney et al., 2016; Sweeney et al., 2018), TriVerity generates two different scores, one to differentiate infection type (e.g., bacterial vs. viral) and another, disease severity. (Liesenfeld et al., 2025). However, validation of TriVerity’s diagnostic accuracy in neonates needs to be further assessed.

An additional issue with biomarker discovery is that there is a growing need not just for diagnostic markers that can identify sepsis, but also predictive markers that can identify which healthy neonates may eventually develop sepsis, thus providing risk stratification, efficient monitoring, and potentially providing earlier care (An et al., 2024). An et al. took advantage of a large vaccination study to analyze a cohort of 700 neonates who had blood collected at birth, among this cohort, 21 neonates developed either EOS or LOS incidentally. Interestingly, neonates who later developed EOS already had ∼1,000 DE genes at birth (when still appearing clinically healthy) when compared to healthy control neonates or those who later developed a localized infection or LOS. Using machine learning methods, a 4-gene signature comprised of HSPH1, BORA, NCAPG2, and PRIM1 was identified that could predict with 93% sensitivity and 92% specificity which neonates would later develop EOS based on blood transcriptomics at birth (An et al., 2024). Such a predictive blood biomarker would be incredibly useful as a screening tool to accurately risk stratify neonates at birth for closer monitoring and earlier treatment of neonatal sepsis. Overall, omics biomarkers may eventually aid early diagnosis and intervention (Liang et al., 2022), ultimately achieving accurate diagnostic at the bedside, and possibly guiding personalized treatment (Iregbu et al., 2022; Kurul et al., 2021).

## Systems immunology identify potential therapeutic pathways

Accurate diagnoses and effective treatment of sepsis can greatly improve outcomes (Demir et al., 2025). However, in addition to the current diagnostic challenges discussed above (e.g., lack of standard case definitions and a lack of sensitive biomarkers), the heterogeneity of disease presentation makes finding new treatments difficult (Jain et al., 2024). Biological variability among sepsis patients influences response to treatment, highlighting the need to better understanding sepsis pathophysiology to design more effective treatment strategies (Demir et al., 2025). For instance, sepsis patients may be categorized into distinct sub-phenotypes to implement more personalized treatment strategies (Demir et al., 2025).

Genome-wide transcriptomic analyses have been used to successfully untangle the patient heterogeneity seen in adult sepsis by clustering them into distinct “endotypes” based on unique immune dysfunction and pathophysiology (Baghela et al., 2022; Scicluna et al., 2025). In pediatric sepsis, unsupervised clustering of whole-blood transcriptome identified three distinct subclasses of pediatric septic individuals, with one cluster associated with younger, sicker patients with a higher mortality rate (Wong et al., 2009). Recently, using publicly available gene-expression datasets and unsupervised clustering with T-distributed Stochastic Neighbor Embedding (t-SNE) Al Gharaibeh (Al Gharaibeh et al., 2025) identified three distinct endotypes of neonatal sepsis, with the mortality endotype having higher cardiac dysfunction and driven by overactive innate immune responses from neutrophil progenitors and repression of T-lymphocyte signaling.

Currently, empiric antibiotics are given immediately to neonates suspected of sepsis (Mukhopadhya et al., 2019; Parkinson et al., 2025). Empiric antibiotic used to treat suspected sepsis holds potential consequences for future health and antimicrobial resistance (AMR) (Parkinson et al., 2025; Stocker et al., 2024). For example, recent work has shown the importance of early-life assembly dynamics in the gut microbiota and how it is prone to external disruptions like early antibiotic use (Gibson et al., 2015). These changes to the gut microbiome persist to later in life as antibiotic resistance gene reservoirs in the microbiota, which negatively influence the efficacy of future antimicrobial therapy (Gibson et al., 2015). The Burden of Antibiotic Resistance in Neonates from Developing Societies (BARNARDS) study was designed to look at the impact of antimicrobial resistance in neonatal sepsis cases from LMICs (Thomson et al., 2021; Sands et al., 2021). The BARNARDS observational cohort spanned across seven LMICs in Africa and South Asia and recorded the frequency of resistance and sepsis mortality for treatment by different antibiotics (Thomson et al., 2021), as well as identified the top 5 bacterial pathogens and performed whole-genome sequencing to detect AMR in the bacterial genomes (Sands et al., 2021). Despite the risk of AMR, clinicians still support the use of antibiotic treatment because of how fast sepsis progresses in newborns and the consequences from delay. However, a more judicious use of antibiotics is needed, and models of care should be adapted to facilitate serial clinical and laboratory assessments in low-risk infants (Lavoie et al., 2019).

Iregbu et al. (2022) highlight the role for multi-omics and big data to avoid misdiagnoses and the associated spread of AMR (Iregbu et al., 2022). Led by clinicians, the publication argues that healthcare systems globally need precision medicine and how this may be more difficult in LMIC settings, where sepsis is most prominent (Iregbu et al., 2022). Hancock et al. (2025), provide an overview on how systems biology incorporating multi-omics, machine learning, and network analyses could be used to improve sepsis diagnostics in adults (Hancock et al., 2025). They suggest that immune-directed therapies made specific to sepsis endotypes may transform sepsis care in the future (Hancock et al., 2025), for example, targeting angiogenesis-associated pathways with interventions that increase Ang-1 activity (Fidanza et al., 2024).

Vascular dysfunction is a key hallmark of sepsis pathophysiology due to multi-system organ failure (Hotchkiss et al., 2016). Specifically, the angiopoietin (Ang)/TIE-2 signaling, which regulates vascular homeostasis, has emerged as a critical pathway underlying the core pathogenesis of sepsis in both adults and newborns (Leligdowicz et al., 2018; Wright et al., 2018). This has led to several recent work showing that the Ang/TIE-2 pathway can serve as a sepsis biomarker as well as therapeutic potential (Fidanza et al., 2024; Chi et al., 2024). Angiopoietin is a family of oligomeric-secreted glycoproteins, and include Ang-1, Ang-2, Ang-3 and Ang-4 (Chi et al., 2024). The two best characterized angiopoietins, Ang-1 and Ang-2, maintains vascular homeostasis where Ang-1 preserves vascular integrity, while Ang-2 promotes angiogenesis by breaking down endothelial intercellular junctions (Chi et al., 2024). This balancing act requires a third player, TIE-2, which is a tyrosine kinase and serves as Ang receptors on endothelial cells. Typically, Ang-1 binds to and phosphorylates TIE-2 receptor to promote vascular homeostasis and integrity. In contrast, Ang-2 is typically sequestered in specialized Weibel-Palade (WP) bodies and are released during inflammatory and oxidative stress; Ang-2 acts as competitive antagonist for the TIE-2 receptor, increasing vascular permeability (Chi et al., 2024).

In adult septic patients, the Ang-2/Ang-1 ratio serves as prognostic biomarkers, with increasing plasma Ang2 concentration and higher Ang-2/Ang-1 ratio associated with increasing sepsis severity (David et al., 2012; Fang et al., 2015). This trend is similarly observed in preterm neonates with sepsis (Fidanza et al., 2024; Leligdowicz et al., 2018). Using a mouse neonatal sepsis model with cecal slurry, Fidanza et al. show in a series of mechanistic experiments that manipulating the Ang-1 vs. Ang-2 ratio can alter survival. Particularly, the survival of septic neonatal mice is dramatically improved when Ang-1 levels increase via prophylactic administration of exogenously Ang-1, while exogenous Ang-2 had little effect on survival (Fidanza et al., 2024). Furthermore, given Ang-2’s role in increasing vascular permeability, prophylactic treatment of anti-Ang-2 blocking antibody to decrease Ang-2 levels led to improved survival. This suggests that Ang-1 could potentially serve a prophylactic or therapeutic intervention in neonatal sepsis.

Using a combination of machine learning and whole-blood transcriptomic analyses, Fidanza et al. show that cecal-slurry challenged neonatal mice that are predicted to survive vs. not-to-survive had differential expression in the arachidonic acid pathways (Fidanza et al., 2024). Low arachidonic acid levels in premature infants have been shown to be associated with increased risk of late-onset sepsis (Martin et al., 2011). In two independent sepsis cohorts, an Australian preterm cohort and a Malawian full-term cohort, critical genes in the arachidonic acid metabolism pathways are dysregulated in the septic infants compared to control (Fidanza et al., 2024). Furthermore, prophylactic treatment with arachidonic acid improved neonatal mice survival, and likely mediate through the Ang/TIE-2 pathway, as shown by decreasing the Ang-2/Ang-1 ratio (Fidanza et al., 2024). Collectively, the identification of this functional link between the arachidonic acid metabolism and regulation of the Ang/TIE-2 pathway provides future directions for potential pathogen-agnostic immunomodulatory therapy for neonatal sepsis.

## Concluding remarks

Neonatal sepsis remains a deadly and difficult disease to diagnose, especially in preterm neonates. The key to understanding their unique vulnerability to sepsis lies in characterizing their developing immune system at the early stages of life, which differs greatly from term neonates and from adults. This review highlighted the complex interplay between the neonatal immune system and its response to sepsis with a focus on the influence of gestational ages. The dynamics of the immune system likely explains the different responses towards infection early in life. Preterm neonates have impaired innate immunity, including reduced PRR responses and lower neutrophil and monocyte functions, compared to term neonates, which may greatly increase their susceptibility to sepsis and bacterial infections in general. During sepsis, neonates experience a simultaneous release of pro-inflammatory and anti-inflammatory cytokines, which, if imbalanced, can lead to excessive inflammation, septic shock, organ dysfunction, and mortality. Nevertheless, the developmental ontogeny of neonates during their first days of life is incompletely understood. The recent boom of high-throughput omics studies has allowed a more thorough understanding of the immune development trajectory of neonates and of the pathways that become disrupted during sepsis. This has allowed a better understanding of the mechanistic underpinnings of neonatal sepsis. Continued advances in our understanding of neonatal sepsis bring us closer to the timely detection and effective treatment of a life-threatening condition that disproportionately affects the youngest and most vulnerable patients, supported by the development of more accurate diagnostic and predictive biomarkers.
